# Transcatheter aortic valve implantation versus surgical aortic valve replacement in patients at low and intermediate risk: A risk specific meta-analysis of randomized controlled trials

**DOI:** 10.1371/journal.pone.0221922

**Published:** 2019-09-24

**Authors:** Fang Fang, Jingjing Tang, Yaqin Zhao, Jialing He, Ping Xu, Andrew Faramand

**Affiliations:** 1 West China Hospital, Sichuan University, Chengdu, Sichuan, China; 2 The Chinese University of Hong Kong, Shenzhen, Guangdong, China; 3 Sichuan University Library, Chengdu, Sichuan, China; 4 University of Pittsburgh Medical Center, Pittsburgh, Pennsylvania, United States of America; Case Western Reserve University School of Medicine, UNITED STATES

## Abstract

**Background:**

Transcatheter aortic valve implantation (TAVI) is an option for treatment for patients with severe aortic stenosis who are at high risk for death with surgical aortic valve replacement (SAVR). It is unknown whether TAVI can be safely introduced to intermediate- and low-risk patients.

**Objective:**

To compare the efficacy and safety of TAVI and SAVR in patients with intermediate- and low-surgical risk.

**Data sources:**

Medline, Embase, and the Cochrane Central Register of Controlled Trials were searched from inception to April 15, 2019.

**Study selection:**

We included randomized controlled trials comparing TAVI with SAVR in patients with intermediate- and low-surgical risk.

**Data extraction:**

Meta-analyses were conducted using random-effects models to calculate risk ratios (RR) with corresponding 95% confidence interval (CI). Two independent reviewers completed citation screening, data abstraction, and risk assessment. Primary outcome was a composite of all-cause mortality or disabling stroke at 12 months.

**Data Synthesis:**

A total of 5 trials randomizing 6390 patients were included. In patients with low risk, TAVI was associated with a significant reduction in the composite of all-cause mortality or disabling stroke compared with SAVR (RR, 0.56; 95%CI, 0.40–0.79; I^2^ = 0%). This benefit was not replicated in patients with intermediate risk (RR, 0.96; 95% CI, 0.80–1.15; I^2^ = 0%). Similar results were seen separately in all-cause mortality and disabling stroke when TAVI was compared with SAVR.

**Conclusion:**

For patients with severe aortic stenosis who were at low risk for death from surgery, TAVI achieved superior clinical outcomes compared to SAVR; however, these benefits were not seen in those with intermediate risk. This information may inform discussions about deciding between SAVR and TAVI for patients with low to intermediate risk separately.

## Introduction

Historically, surgical aortic valve replacement (SAVR) was the standard of treatment for patients with severe aortic stenosis.[1] The increased operator experience combined with the technical advances in the new generation valves, transcatheter aortic valve implantation (TAVI) has rapidly become the treatment of choice for patients with inoperable and high-risk severe, symptomatic aortic stenosis.[2] Almost 80% of patients undergoing SAVR are classified as being low to intermediate surgical risk patients. As such, there is a growing interest in comparative studies of TAVI and SAVR in this patient population.[3, 4] The 2017 ACC/AHA guidelines for the management of patients with valvular heart disease have modified the recommendations for TAVR.[2] TAVR is now considered a reasonable alternative to SAVR for the intermediate surgical risk group (recommendation class IIa, level of evidence B-R). For patients with low risk, however, the evidence of whether TAVI may be an alternative to SAVR remains insufficient. Previous meta-analyses generally suggested that TAVI may have similar clinical outcomes for patients with low to intermediate risk compared with SAVR.[5–8] However, those reviews did not assess the effect in patients with intermediate and low risk separately. Since those reviews, the results of several large-scale randomized trials have been reported, with inconsistent conclusions.[9–11] To better understand the impact of surgical risk on the response to TAVI, we performed a risk-specific meta-analysis comparing clinical outcomes of patients with severe aortic stenosis undergoing either TAVI or SAVR.

## Methods

### Protocol and guidance

The study protocol followed the PRIMA-P guidelines[12]. This article has been reported in accordance with the systematic review following Preferred Reporting Items for Systematic Reviews and Meta-Analyses (PRISMA) guidelines[13]. PRISMA checklist is reported in S2 File.

### Eligibility criteria

Eligible studies met the following PICOS (participants, interventions, comparators, outcomes, and study design) criteria:

(1) Population: The population of interest included low- or intermediate-risk adults (age≥18) with severe aortic stenosis. Intermediate risk was defined as the Society of Thoracic Surgeons Predicted Risk of Mortality (STS) score 4–8%, while low-risk was defined as an STS score of less than 4%. Logistic EuroSCORE was used only if an STS score was not available (Intermediate risk with logistic EuroSCORE 10–20%, low risk with logistic EuroSCORE <10%).

(2) Intervention: TAVI.

(3) Comparison intervention: SAVR

(4) Outcome: The primary outcome was a composite of death from any cause or disabling stroke at 12 months. Secondary outcomes were all-cause mortality, disabling stroke, transient ischemic attack, life-threatening or disabling bleeding, acute kidney injury stage 2 or 3, atrial fibrillation, myocardial infarction, and endocarditis. The time frame of all outcomes was 12 months.

(5) Study design: Randomized controlled trials

Exclusion Criteria. (1) case reports, case series, and observational studies, (2) trials that compared TAVI with medical therapy or no treatment, (3) trials that patients were treated by different devices of TAVI, (4) trials with less than one-year follow-up.

### Data sources

The search strategy was developed by a medical librarian (PX). The databases Medline, Embase, and the Cochrane Central Register of Controlled Trials were searched from inception to April 15, 2019. We also checked the reference lists of included trials and reviews for additional studies. We searched trial registries on ClinicalTrials.gov for ongoing studies or the availability of completed studies with reported results. We did not use any language restrictions. The details of the search strategy conducted are presented in Table A in S1 File.

### Study selection

After removal of duplicates, two authors (FF and JH) independently screened the title and abstracts of the search results. The full texts of the remaining papers were assessed in independently by the two authors. Disagreements between the two authors required resolution by a third reviewer.

### Data collection process

Two authors (FF and JH) independently extracted data about study characteristics and event rates from the eligible trials into standardized collection forms. Disagreements between the two authors required resolution by a third reviewer.

### Risk of bias, publication bias, and quality of evidence

Two authors (FF and JH) independently performed risk of bias assessment using the Cochrane Collaboration risk of bias tool across five domains (sequence generation, allocation concealment, blinding, detection bias, and attrition bias).[14] Each domain was assessed as either low, unclear, or high risk of bias. Disagreements between the two authors required resolution by a third reviewer. Two authors (FF and JH) independently rated the confidence in the estimates of effect for each outcome using the Grading of Recommendations, Assessment, Development and Evaluation (GRADE).[15] We assessed the small study effects using a visual estimate of the funnel plot and using the regression test of Egger’s test, Begg’s test, and Harbord’test when 10 or more trials were pooled.[16]

### Data synthesis

All statistical analyses were performed using RevMan (5.3.3; The Cochrane Collaboration). Analyses for all outcomes were done on an intention-to-treat basis. Pooled effect sizes were calculated using a random-effects model. Dichotomous variables were analyzed using the Mantel-Haenszel method and were expressed as risk ratios (RR). A P value of < 0.05 was set for statistical significance. Heterogeneity was assessed using with the I^2^ test, with I^2^ > 50% being considered substantial.[17]

Variables for subgroup analysis were identified based on patients with low and intermediate risk. For trials involving mixed risk patients but not presenting separate data, we included the pooled results in the low/intermediate-risk patients subgroup only if ≥ 80% of patients were low/intermediate -risk. Otherwise, we excluded the trials form subgroup analysis. Moreover, we performed a meta-regression subgroup analysis to test the interaction between surgical risk and the magnitude of effect using an STS score as a predictor.

Sensitivity analyses were conducted for the primary outcome by (1) excluding trials at each time, (2) using fixed-effect models, (3) excluding trials with a non-low risk of bias except performance bias, (4) excluding trials with less than 1000 patients, (5) excluding trials with early generation valve.

## Results

### Study selection and study characteristics

**Fig 1:**
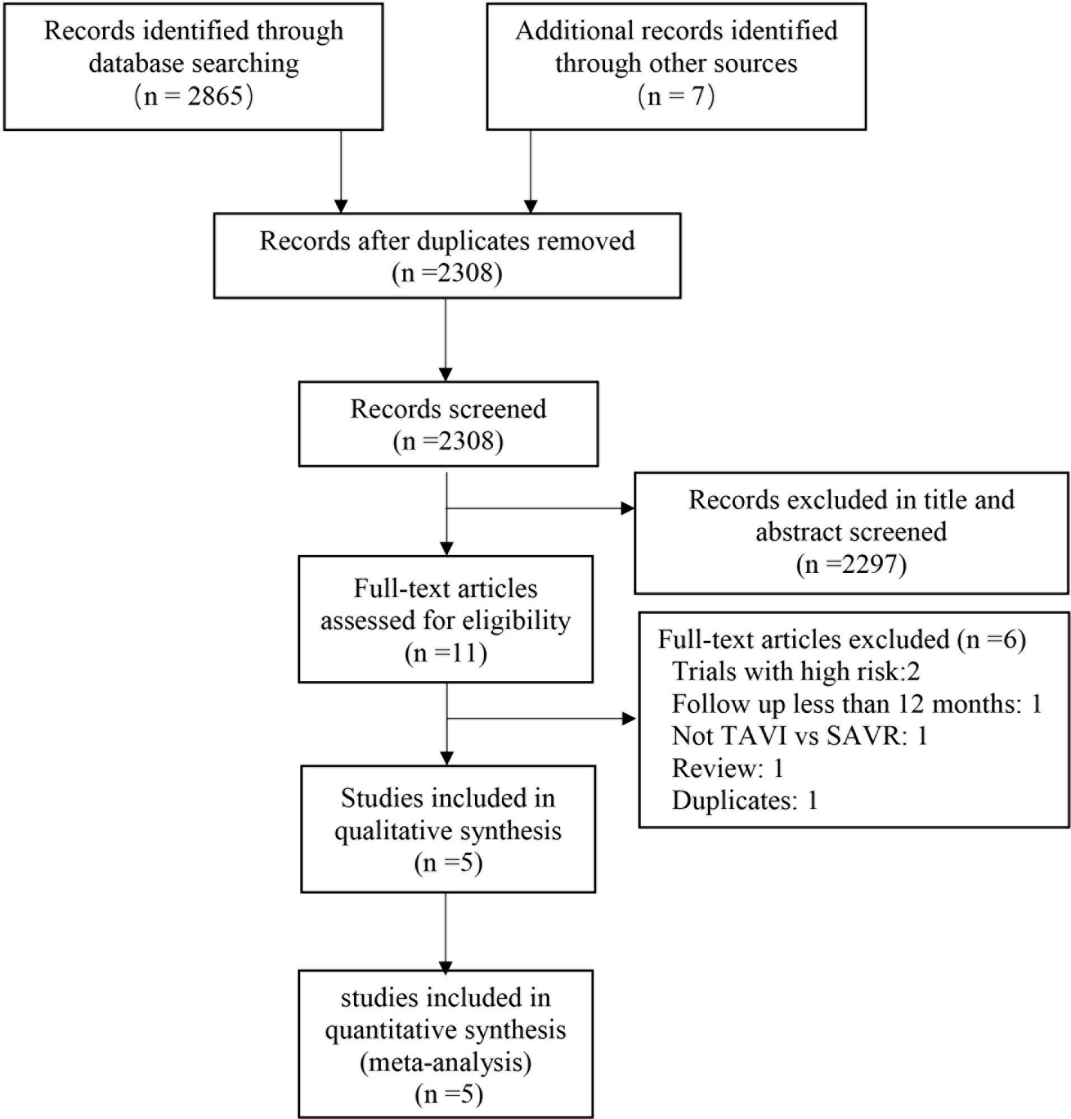
Search strategy and final included and excluded studies.

Details of the study selection process are presented in Fig 1. Of the 2872 results, five trials[9–11, 18, 19] with a total of 6390 patients were included in the final meta-analysis. Studies were excluded for the following reasons: two studies included patients with high risk, 1 have follow-up less than 1 year, one was not comparison of TAVI and SAVR, one was review, and one was duplicate (Table B in S1 File).

The key characteristics of all included studies are available in Table 1. The studies were published between 2012 and 2019. Population sizes ranged from 280 to 2032 patients. All trials were multicenter. Three trials included patients with low risk[10, 11, 18], and one trial studied patients with intermediate risk. A post hoc analysis of the SURTAVI[9, 20] presented subgroups of low and intermediate risk patients. The definitions of surgical risk varied slightly across studies, which were presented in Table C in S1 File.

**Table 1:** Characteristics of Included Studies.

	Number of centers	Recruitment period	No. of patients	TAVI valve system	Age, mean (SD)	Women, %	STS, mean (SD)		Follow-up, year
							Low risk	Intermediate risk	
NOTION ^18^	3	2009–2013	280	CoreValve	79.1±4.8	53.20	3.0±1.6	NR	5
Evolut R ^11^	86	2016–2018	1468	Evolut R, Corevalve, or Evolut PRO	73.9±6.0	34.9	1.9±0.7	NR	2
PARTNER 3 ^10^	71	2016–2017	1000	SAPIEN 3	73.3±5.8	65.8	1.9±0.6	NR	1
PARTNER 2A ^19^	57	2011–2013	2032	SAPIEN XT	81.6±6.7	54.5	NR	5.8±2.0	2
SURTAVI ^9^	87	2012–2016	1746	Evolut R or Corevalve	79.6±6.1	43.2	2.3±0.5;	4.9±0.8	2

NR: not reported

### Risk of bias, publication bias, and quality of evidence

Risk-of-bias assessments are reported in Figs A and B in S1 File. The main bias was due to the lack of blinding to intervention in both participants and personnel. Key findings of GRADE assessment of certainty for main outcomes are shown in Table D in S1 File. We did not perform analysis to detect small study effects because of the small number of studies included.

### Primary outcome

**Fig 2:**
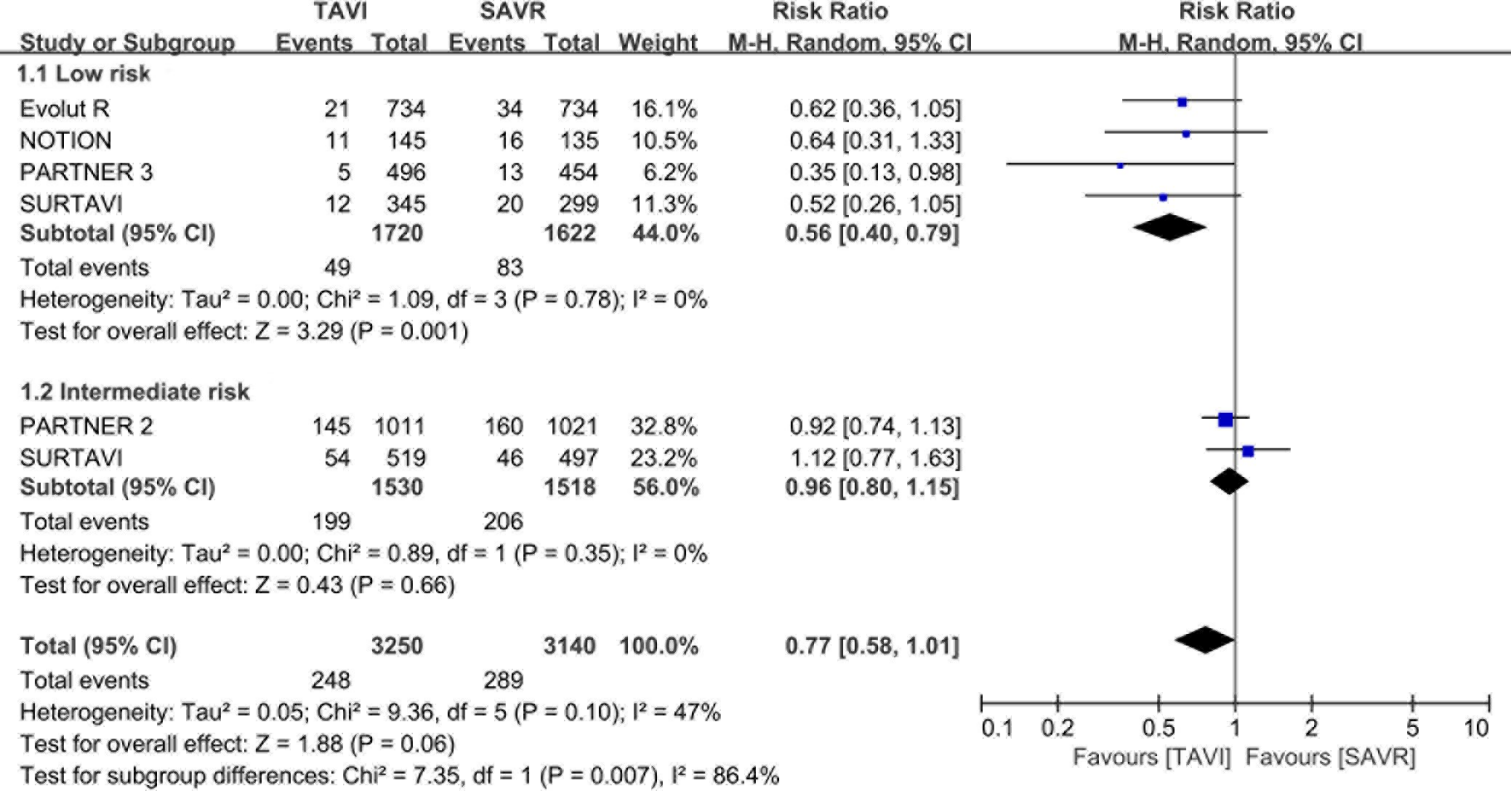
Association of TAVI vs SAVR with a composite of all-cause mortality and disabling stroke, stratified by surgical risk. TAVI = Transcatheter aortic valve implantation, SAVR = surgical aortic valve replacement, M-H = Mantel-Haenszel.

**Fig 3:**
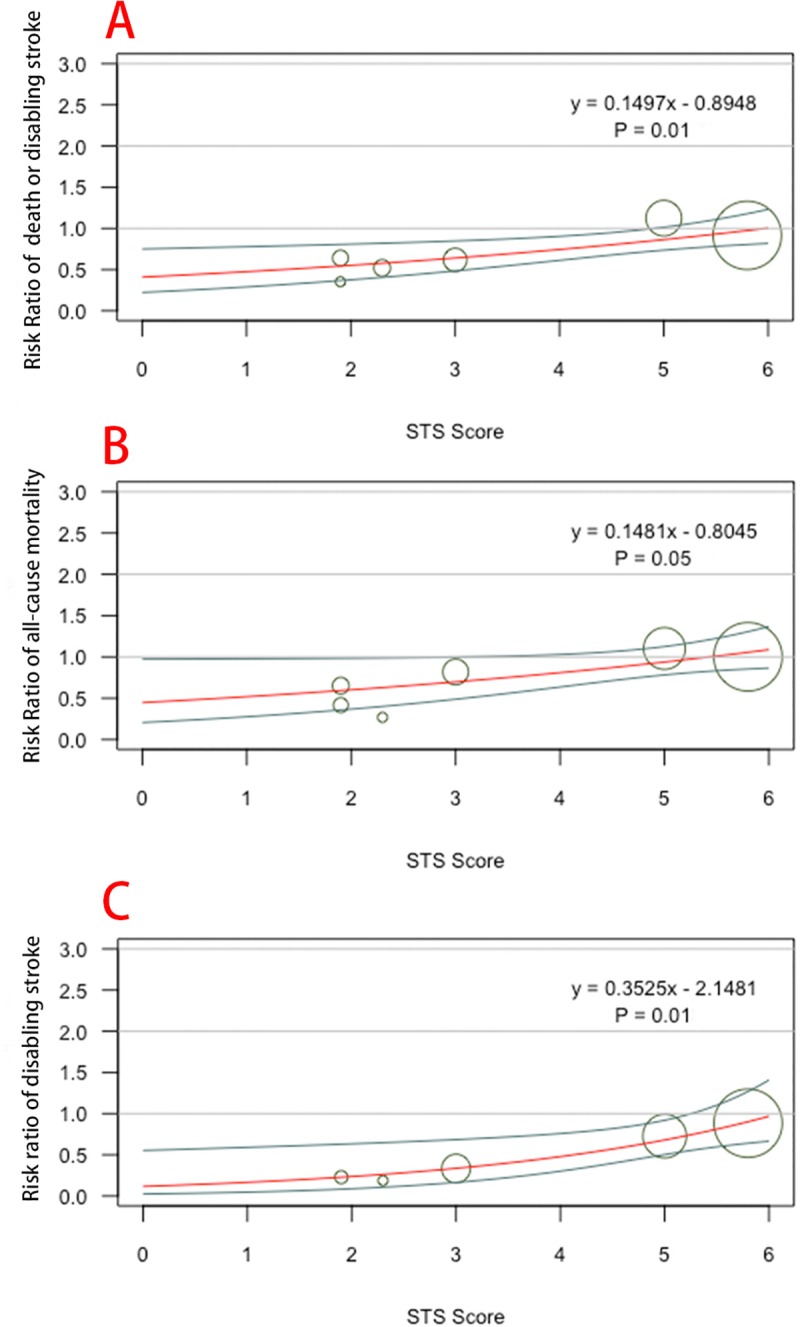
Interaction between an STS score and survival/stroke benefits with TAVI. Meta-regression analysis confirmed the positive interaction between an STS score and the benefits of TAVI, with lower STS score with (A) a composite of all-cause mortality and disabling stroke (P = 0.0135), (B) all-cause mortality (P = 0.05), and (C) lower cumulative doses (P = 0.01). STS = Society of Thoracic Surgeons Predicted Risk of Mortality;TAVI = transcatheter aortic valve implantation.

All five eligible trials reported the composite of all-cause mortality or disabling stroke at 12 months (the primary outcome). In patients with low risk, the incidence of all-cause mortality or disabling stroke at 12 months for TAVI vs SAVR was 49 of 1720 (2.8%) vs 83/1622 (5.1%) (RR, 0.56; 95%CI, 0.40–0.79; I^2^ = 0%; Fig 2). In those with intermediate risk, the risk of all-cause mortality or disabling stroke at 12 months for TAVI vs SAVR was 199 of 1530 (13.0%) vs 206/1518 (13.6%) (RR, 0.96; 95% CI, 0.80–1.15; I^2^ = 0%). The subgroup analysis of primary outcome revealed heterogeneity between patients with low and intermediate risk (P = 0.007). Moreover, meta-regression analysis confirmed the positive interaction between an STS score and the primary outcome, with a lower RR of the composite of all-cause mortality or disabling stroke at a lower STS score (P = 0.01; Fig 3).

### Secondary outcomes

**Fig 4:**
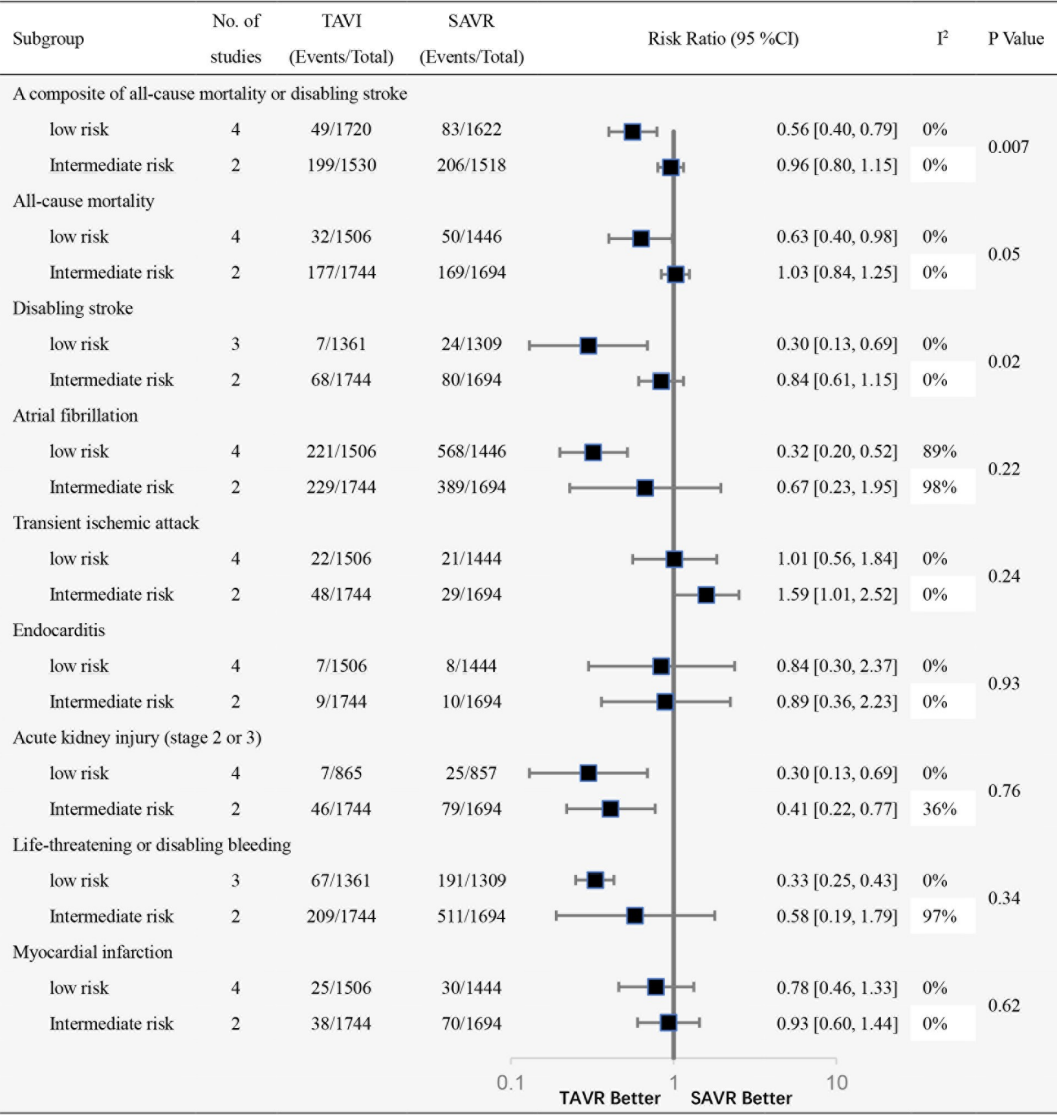
Summary of findings for outcomes in the review, stratified by patients with intermediate and low risk. TAVI = transcatheter aortic valve implantation; SAVR = surgical aortic valve replacement.

The summary of all outcomes is presented in Fig 4; the forest plots of these outcomes are shown in Fig C-J in S1 File. Similar with primary outcome, TAVI was associated with significant benefit for all-cause mortality (RR, 0.63; 95% CI, 0.40–0.98) in patients with low risk but not in patients with intermediate risk (RR, 1.03; 95% CI, 0.84–1.25), with substantial heterogeneity across trials based on surgical risk (P = 0.05; I^2^ = 74.5%). Among patients with low risk, TAVI was associated with a 70% reduction in the risk of disabling stroke (RR, 0.30; 95% CI, 0.13–0.69). In patients with intermediate risk, however, there was no difference in the risk of disabled stroke between treatment groups (RR, 0.84; 95% CI, 0.61–1.15). The subgroup analysis of disabling stroke showed substantial heterogeneity across trials (P = 0.02). Meta-regression analysis also confirmed the positive interaction between an STS score and disabling stroke.

TAVI was associated with a significant reduction in the risk of acute kidney injury stage 2 or 3 in both risk groups. Among patients with low risk, TAVI was associated with a significant reduction in atrial fibrillation and life-threatening or disabling bleeding, but those benefits were not seen in patients with intermediate-risk.

### Sensitivity analysis

Similar results were observed for the composite of all-cause mortality or disabling stroke at 12 months in all conducted sensitivity analyses by (1) excluding trials at each time, (2) using fixed-effect models, (3) excluding trials with non-low risk of bias except performance bias, (4) excluding trials with less than 1000 patients, (5) excluding trials with early generation valve (Table E in S1 File).

## Discussion

In this risk-specific meta-analysis of 5 trials with a total of 6390 patients, we found that the benefits of TAVI compared with SAVR were different between patients with low and intermediate risk. In patients with severe aortic stenosis who were at low risk, TAVI was associated with a reduced rate of the composite of all-cause mortality and disabled stroke at 12 months compared with SAVR. In contrast, in those with intermediate risk, TAVI did not affect the incidence of the composite of all-cause mortality and disabled stroke compared with SAVR. This risk-specific difference was also seen in other important outcomes, such as disabling stroke and all-cause mortality. Moreover, prespecified subgroup analyses revealed heterogeneity between patients with low and intermediate risk, and meta-regression demonstrated an opposite interaction of STS score with the composite of all-cause mortality and disabled stroke when TAVI compared with SAVR.

### Comparison with other studies

Our findings are not consistent with those from previous meta-analyses.[5–8, 21, 22] Those studies found that TAVI and SAVR may have similar all-cause mortality and stroke rates in low- and intermediate-risk patients.[5–8, 21, 22] Further, our study documented differences between low and intermediate risk patients in the credibility of the crucial outcomes(death and disabled stroke). This difference in part may be explained by the inclusion two trials of patients with low risk[10, 11], a feature that accounted for 89.6% (2418/2698) of the total number of low-risk patients. This data improved the precision concerning the treatment effects and provided enough power to find a subgroup difference. Moreover, we identified some new findings, including a decreased risk of atrial fibrillation and life-threatening or disabling bleeding with TAVI only in patients with low risk.

### Strengths and limitations

This meta-analysis summarized the findings of 5 trials, including a total of 6390 patients. This produces more robust estimates than previous studies. Our approach included a comprehensive search strategy, explicit eligibility criteria, and a rigorous use of the GRADE evaluations of the quality of evidence on important outcomes. Our meta-analyses included a rigorous assessment of the credibility of subgroup analyses (with crucial differences between low- and intermediate-risk patients).

Several limitations must be considered. First, definitions of surgical risk varied across studies. There were four trials including low-risk patients. Two trials defined the low-risk as an STS score<3%[9, 11], whereas the other two defined low risk as an STS score<4%[10, 18]. As we lacked patient-level data, we could not assess the heterogeneity. Second, this study included few trials in any one comparison to be able to adequately evaluate the bias of small study effects. However, the potential bias of small study effects may be low risk because most included trials had negative results. Third, long-term outcomes were not studied in this meta-analysis, because the long-term data is limited. In future studies, long-term follow-up is required to examine whether larger differences in mortality and stroke between TAVI vs SAVR will emerge over time. Fourth, both early- and new- generation transcatheter heart valve systems were used in the included trials. Better results may be associated with new-generation TAVI devices.[23] Thus, the unbalance of TAVI devices used in trials may affect the results. However, we conducted a sensitivity analysis excluding trials of early generation valve, which did not change the current evidence.

### Implications

During the past decade, the technical advances of the new generations of transcatheter heart valves have broadened the indications for TAVI from high-risk of surgery patients to lower risk patients as an alternative to SAVR.[8, 23] However, current guidelines do not support the use of TAVI in patients with low risk and younger patients, for whom SAVR is standard therapy.[2] These recommendations limited hard evidence of patients with low-surgical risk. The present meta-analysis of all data from randomized trials, in which evaluating the new generation of valves, provided evidence which suggested that TAVI was associated with reduced all-cause mortality or disabled stroke compared with SAVR. This new evidence should lead us to reconsider these recommendations. Moreover, new generation devices have been continually changing, which may proceed to renew the current evidence.

## Conclusions

For patients with low surgical risk, TAVI reduced the risk of all-cause mortality, disabled stroke, and a composite of all-cause mortality or disabled stroke at 12 months. However, these benefits were not seen in patients with intermediate surgical risk.

## Supporting information

S1 File Supplement e-material. (DOCX)

S2 File PRISMA 2009 checklist. (DOCX)
