# Supplementary material for: Transcatheter aortic valve implantation versus surgical aortic valve replacement in patients at low and intermediate risk: A risk specific meta-analysis of randomized controlled trials
Source: PLoS One. 2019 Sep 24;14(9):e0221922. doi: 10.1371/journal.pone.0221922 (PMC6759164; doi:10.1371/journal.pone.0221922)
Supplement: S1 File — (DOCX) [file pone.0221922.s001.docx]

File. Supplement e-material

| Table A: Search strategy |  |
| --- | --- |
| Table B: Excluded trials and reason |  |
| Table C: Definitions of low and intermediate risk across studies |  |
| Table D: Summary of Findings and Strength of Evidence (GRADE) |  |
| Table E: Sensitivity analyses |  |
| Fig A: Risk of bias summary |  |
| Fig B: Risk of bias graph |  |
| Fig C: Forest plot for all-cause mortality |  |
| Fig D: Forest plot for disabling stroke |  |
| Fig E: Forest plot for atrial fibrillation |  |
| Fig F: Forest plot for transient ischemic attack |  |
| Fig G: Forest plot for endocarditis |  |
| Fig H: Forest plot for acute kidney injury stage 2 or 3 |  |
| Fig I: Forest plot for life-threatening or disabling bleeding |  |
| Fig J: Forest plot for myocardial infarction |  |
|  |  |
|  |  |
|  |  |
|  |  |
|  |  |
|  |  |
|  |  |
|  |  |
|  |  |
|  |  |
|  |  |
|  |  |
|  |  |
|  |  |

### Table A: Search strategy

| **MEDLINE(R)** | | |
| --- | --- | --- |
| 1 | exp Aortic Valve Stenosis | 38751 |
| 2 | Aortic Stenosis.mp | 14537 |
| 3 | 1 and 2 | 11909 |
| 4 | (aortic valve implantation or TAVR or transcatheter or transfemoral or transapical or transaxillary or SAVR or heart valve replacement or surgical aortic valve replacement or surgical AVR or SAVR or TAVI or aortic valve replacement or transvascular).af | 34170 |
| 5 | randomized controlled trial.pt. | 479112 |
| 6 | controlled clinical trial.pt. | 92986 |
| 7 | exp randomized controlled trial/ | 479618 |
| 8 | (random*or blind* or trial*). af | 1471732 |
| 9 | 5 or 6 or 7 or 8 | 1471732 |
| 10 | 3 and 4 and 9 | 705 |
| 11 | exp animals/ not humans.sh | 4566440 |
| 12 | 10 not 11 | 703 |
| **Embase** | | |
| 1 | exp Aortic Valve Stenosis/ or Aortic Stenosis.mp | 27785 |
| 2 | (aortic valve implantation or TAVR or transcatheter or transfemoral or transapical or transaxillary or SAVR or heart valve replacement or surgical aortic valve replacement or surgical AVR or SAVR or TAVI or aortic valve replacement or transvascular).af | 82142 |
| 3 | randomized controlled trial.af. | 710190 |
| 4 | controlled clinical trial.af. | 480761 |
| 5 | exp randomized controlled trial/ | 542203 |
| 6 | (random*or blind* or trial*). af | 2288700 |
| 7 | 3 or 4 or 5 or 6 | 2288700 |
| 8 | 1 and 2 and 7 | 1906 |
| 9 | exp animal/ | 23895646 |
| 10 | human/ | 19341774 |
| 11 | 9 not 10 | 4553872 |
| 12 | 8 not 11 | 1903 |
| **Cochrane CENTRAL** | | |
| 1 | exp Aortic Valve Stenosis/ | 737 |
| 2 | (Aortic stenosis OR aorta stenosis OR Aortic Valve Stenoses OR aortic valve stenosis). mp | 1331 |
| 3 | 1 and 2 | 561 |
| 4 | (aortic valve implantation OR heart valve implantation OR TAVR OR TAVI OR transcatheter OR transfemoral OR transapical OR transaxillary OR SAVR OR heart valve replacement OR surgical aortic valve replacement OR surgical AVR OR SAVR).mp. | 2513 |
| 5 | 3 and 4 | 259 |

### Table B: Excluded trials and reason

| **Trial** | **Reason for exclusion** |
| --- | --- |
| Smith 2011^1^ | Trial with high risk |
| Nielsen 2012^2^ | Follow-up less than 12 months |
| Adams 2014^3^ | Trial with high risk |
| Feldman 2018^4^ | Not transcatheter versus Surgical Aortic-Valve Replacement |
| Tarantini 2018^5^ | Review |
| Reardon 2017^6^ | Duplicates, new results published in 2018 7 |

### Table C: Definitions of low and intermediate risk across studies

| PARTNER 2 | Intermediate risk | Patients with STS score 4%-8% and patients with an STS score < 4% when there were coexisting conditions that were not represented in the risk model. |
| --- | --- | --- |
| SURTAVI | Low-risk | Patients with an STS score <3% |
|  | Intermediate risk | Patients with an STS score ≥3% (3% of patients with an STS score>8%) |
|  | Low-risk | Patients with an STS score <3% |
| NOTION | Low-risk | Patients with an STS score <4% (18.2% of patients with an STS score>4%) |
| PARTNER 3 | Low-risk | Patients with an STS score <4% |
| Evolut R | Low-risk | Patients with an STS score <3% |

### Table D: Summary of Findings and Strength of Evidence (GRADE)

| **Outcome** | **No. of patients**  **(Studies)** | Risk ratio **(95% CI)** | **Absolute effect estimates**  **(per 1000)** | | | **Quality** |
| --- | --- | --- | --- | --- | --- | --- |
|  |  |  | **SAVR** | **TAVI** | **Difference** |  |
| A composite of All-cause mortality or disabling stroke at 12 months | | | | | | |
| low risk | 2698(3) | 0.56 [0.40, 0.79] | 48 | 27 | -20 (−29 to -7) | High |
| Intermediate risk | 3048(2) | 0.96 [0.80, 1.15] | 136 | 131 | -5 (−27 to 20) | High |
| All-cause mortality at 12 months | | | | | | |
| low risk | 2952(4) | 0.63 [0.40, 0.98] | 35 | 22 | -13 (−21 to -1) | Moderate^1^ |
| Intermediate risk | 3438(2) | 1.03 [0.84, 1.25] | 100 | 103 | 3 (-16 to 25) | High |
| Disabling stroke at 12 months | | | | | | |
| low risk | 2672(3) | 0.30 [0.13, 0.69] | 18 | 5 | -13 (-16 to -6) | Moderate^1^ |
| Intermediate risk | 3438(2) | 0.84 [0.61, 1.15] | 47 | 39 | -8 (−18 to 7) | High |
| Atrial fibrillation at 12 months | | | | | | |
| low risk | 2952(4) | 0.32 [0.20, 0.52] | 393 | 126 | -267 (−314 to -189) | Low^1,2^ |
| Intermediate risk | 3438(2) | 0.67 [0.23, 1.95] | 230 | 156 | -73 (−342 to 205) | Low^1,2^ |
| Acute kidney injury (stage 2 or 3) at 12 months | | | | | | |
| low risk | 1722(2) | 0.30 [0.13, 0.69] | 29 | 9 | -20 (−25 to -9) | Moderate^1^ |
| Intermediate risk | 3438(2) | 0.41 [0.22, 0.77] | 47 | 26 | -21 (−31 to 5) | Moderate^1^ |

CI: Confidence interval; RR: Risk ratio;

^1^ imprecisions

^2^ inconsistency

### Table E: Sensitivity analyses

|  | Low risk | Intermediate risk |
| --- | --- | --- |
|  | RR, 95% CI | RR, 95% CI |
| Using fixed-effect models | 0.56 [0.39, 0.78] | 0.96 [0.80, 1.15] |
| Excluding trials with non-low risk of bias except performance bias | 0.56 [0.40, 0.79] | 0.96 [0.80, 1.15] |
| Excluding trials with less than 1000 patients | 0.55 [0.34, 0.88] | 0.96 [0.80, 1.15] |
| Excluding trials with early generation valve | 0.35 [0.13, 0.98] | 0.92 [0.74, 1.13] |
| Excluding trials at each time |  |  |
| NOTION | 0.54 [0.36, 0.80] | 0.96 [0.80, 1.15] |
| Evolut R | 0.52 [0.33, 0.82] | 0.96 [0.80, 1.15] |
| PARTNER 3 | 0.59 [0.41, 0.86] | 0.96 [0.80, 1.15] |
| PARTNER 2 A | 0.56 [0.40, 0.79] | 1.12 [0.77, 1.63] |
| SURTAVI | 0.57 [0.39, 0.85] | 0.98 [0.93, 1.02] |

### e Figure A: Risk of bias summary: review authors' judgements about each risk of bias item for each included study.


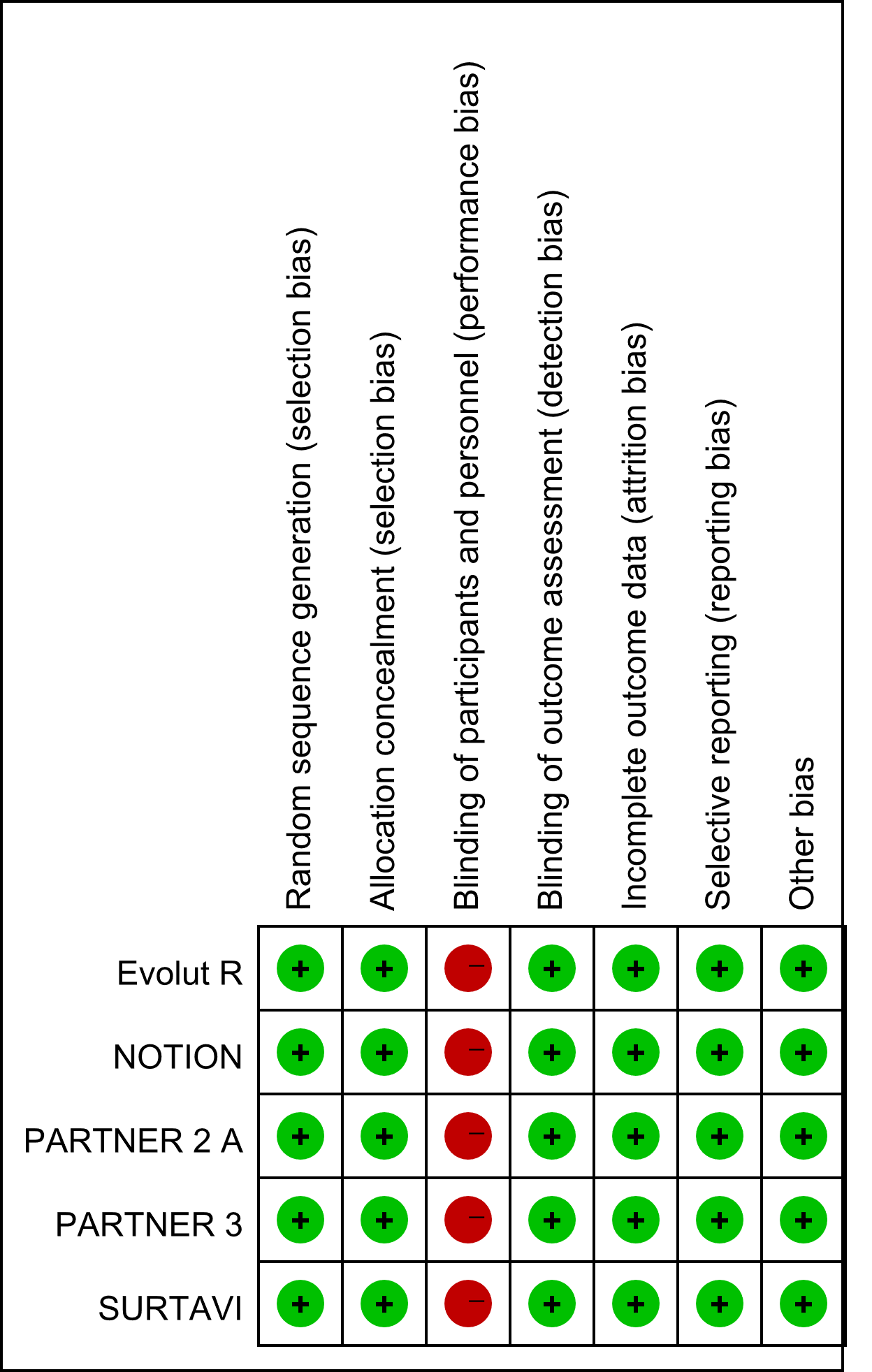


### e Figure B: Risk of bias graph: review authors' judgements about each risk of bias item presented as percentages across all included studies


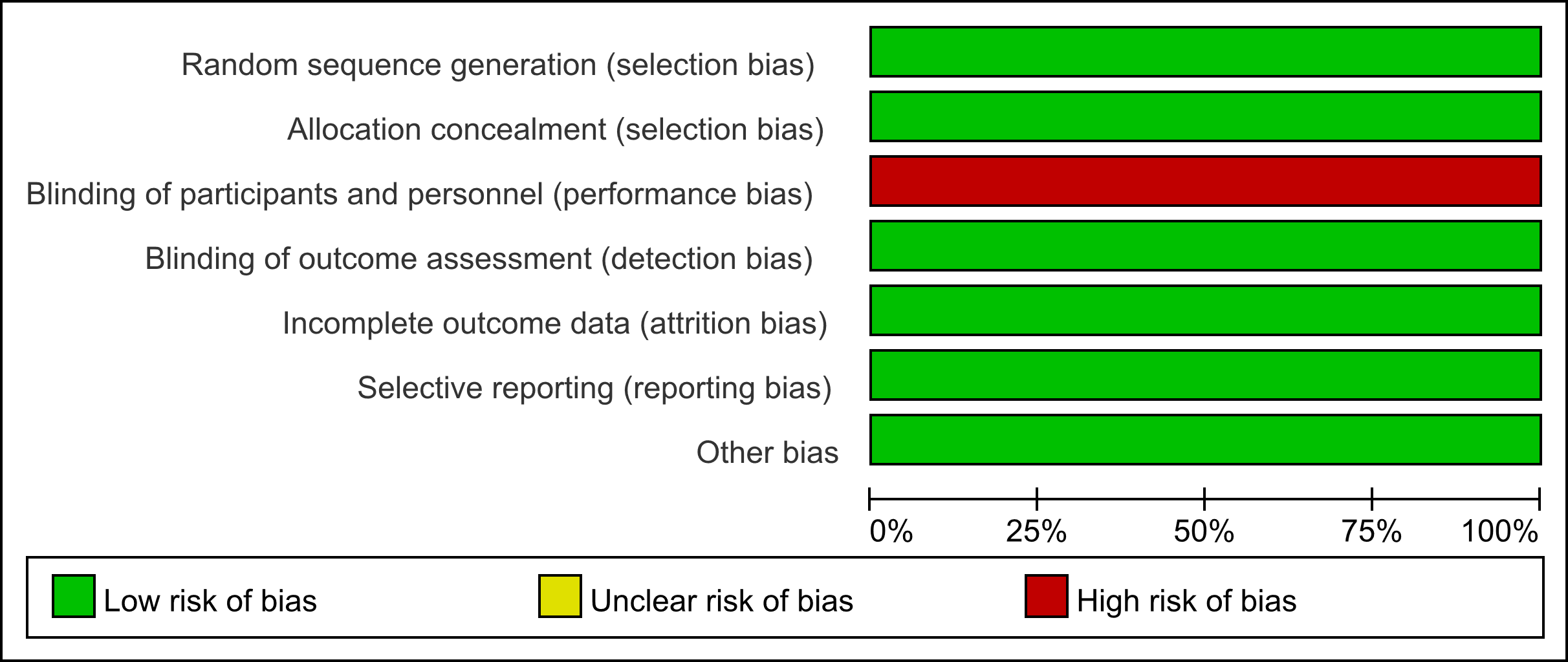


### Fig C: Forest plot for all-cause mortality


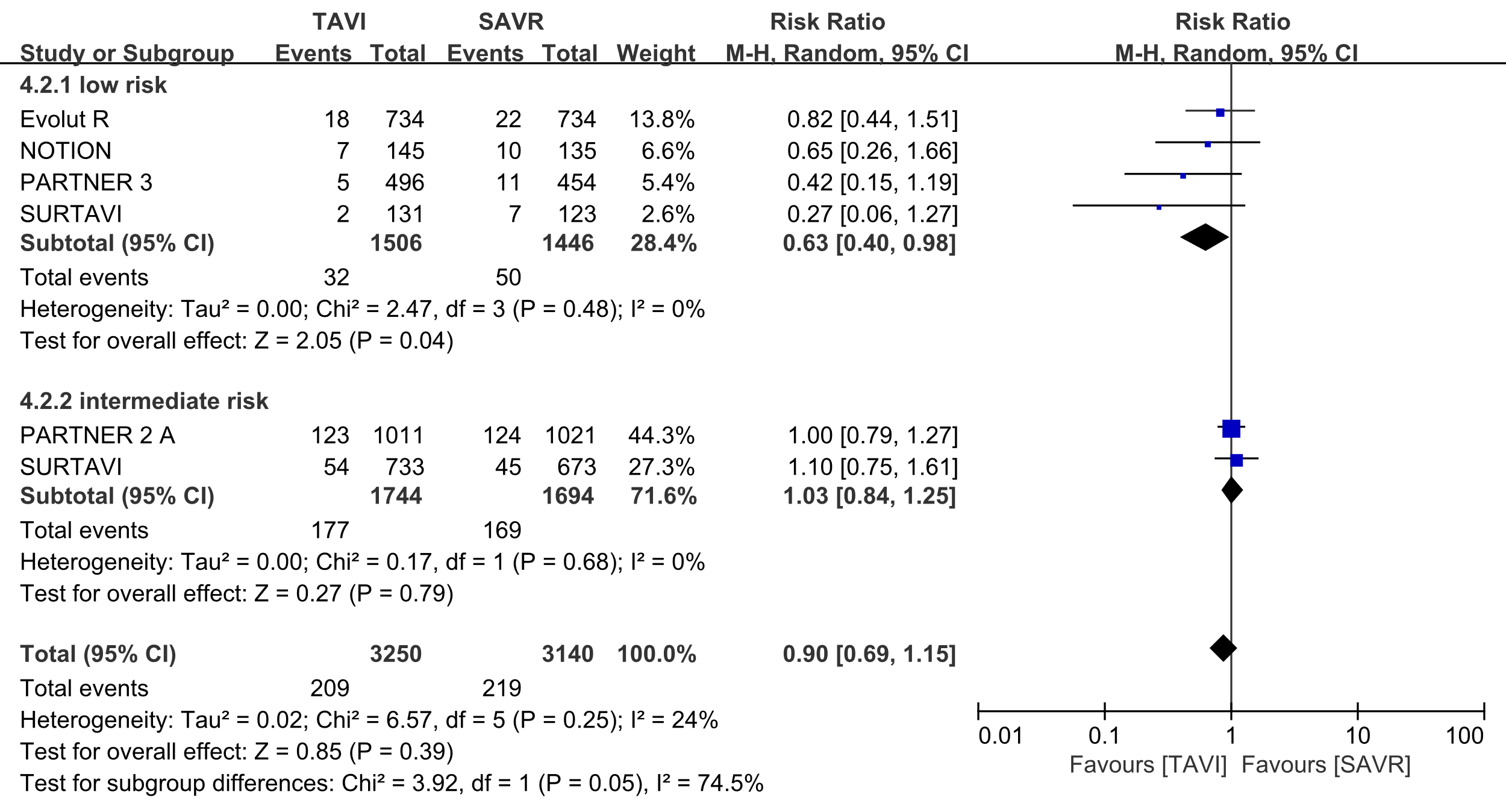


### e Fig D: Forest plot for disabling stroke


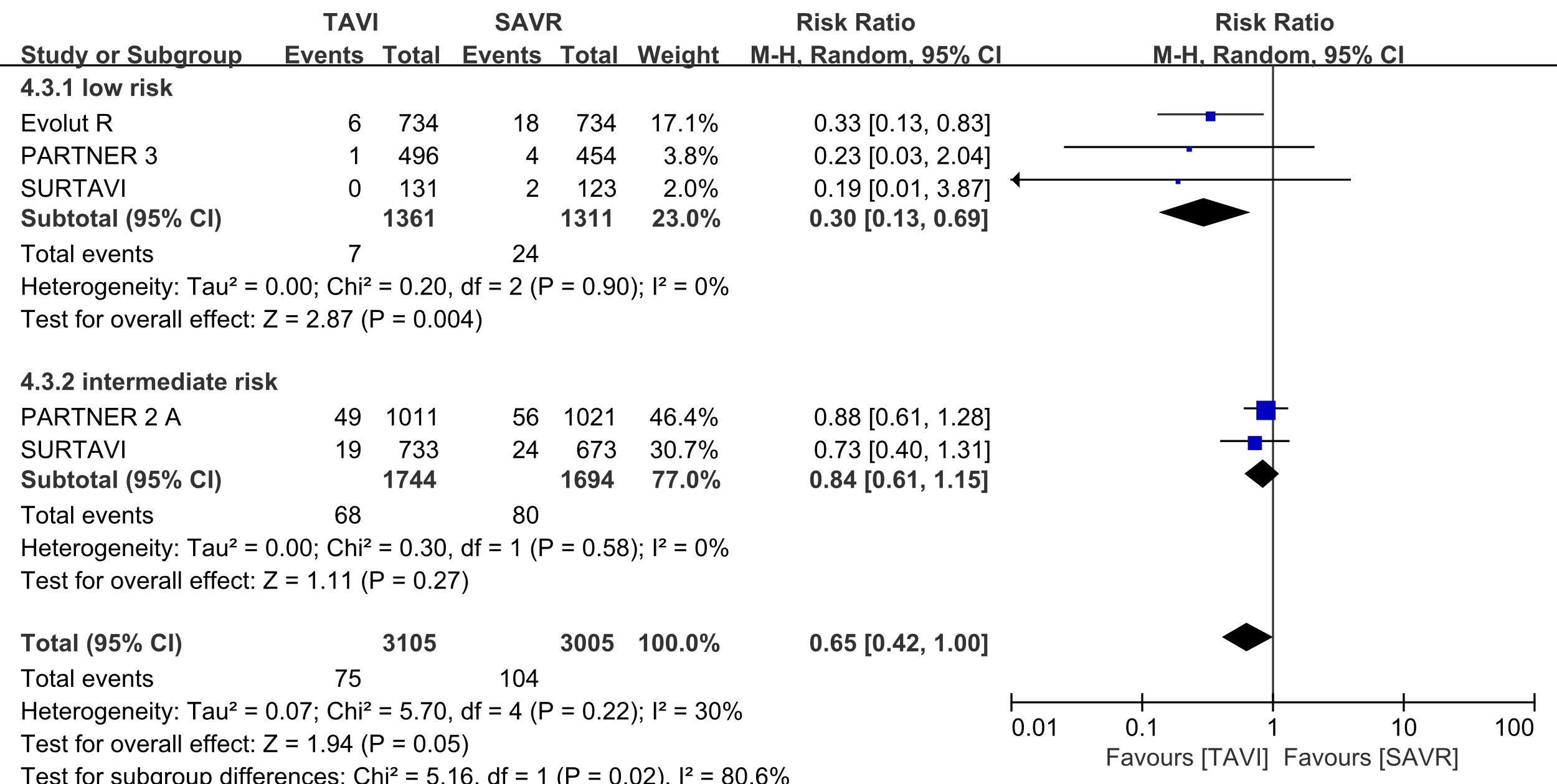


### Fig E: Forest plot for atrial fibrillation

###
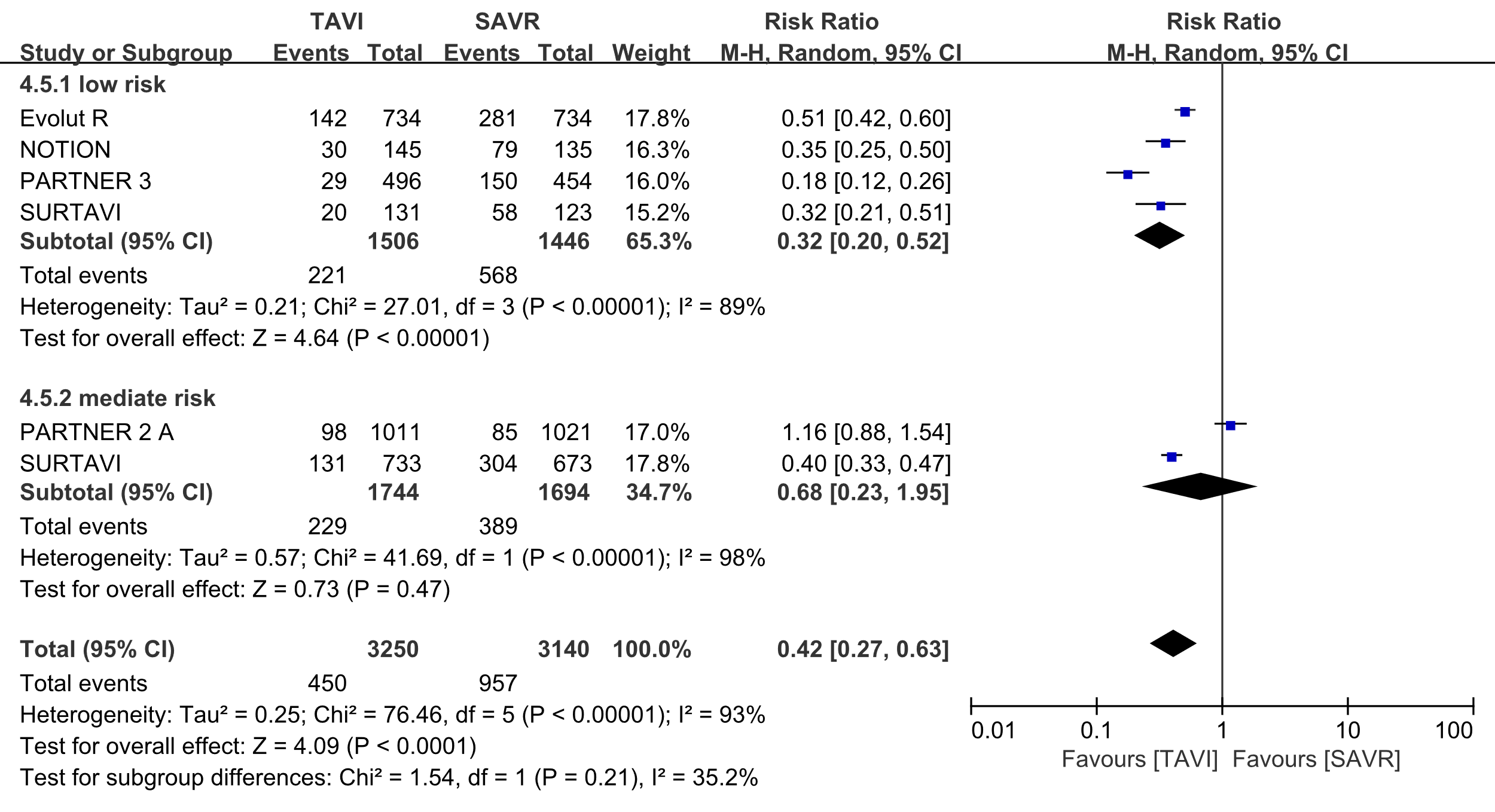


### Fig F: Forest plot for transient ischemic attack

###
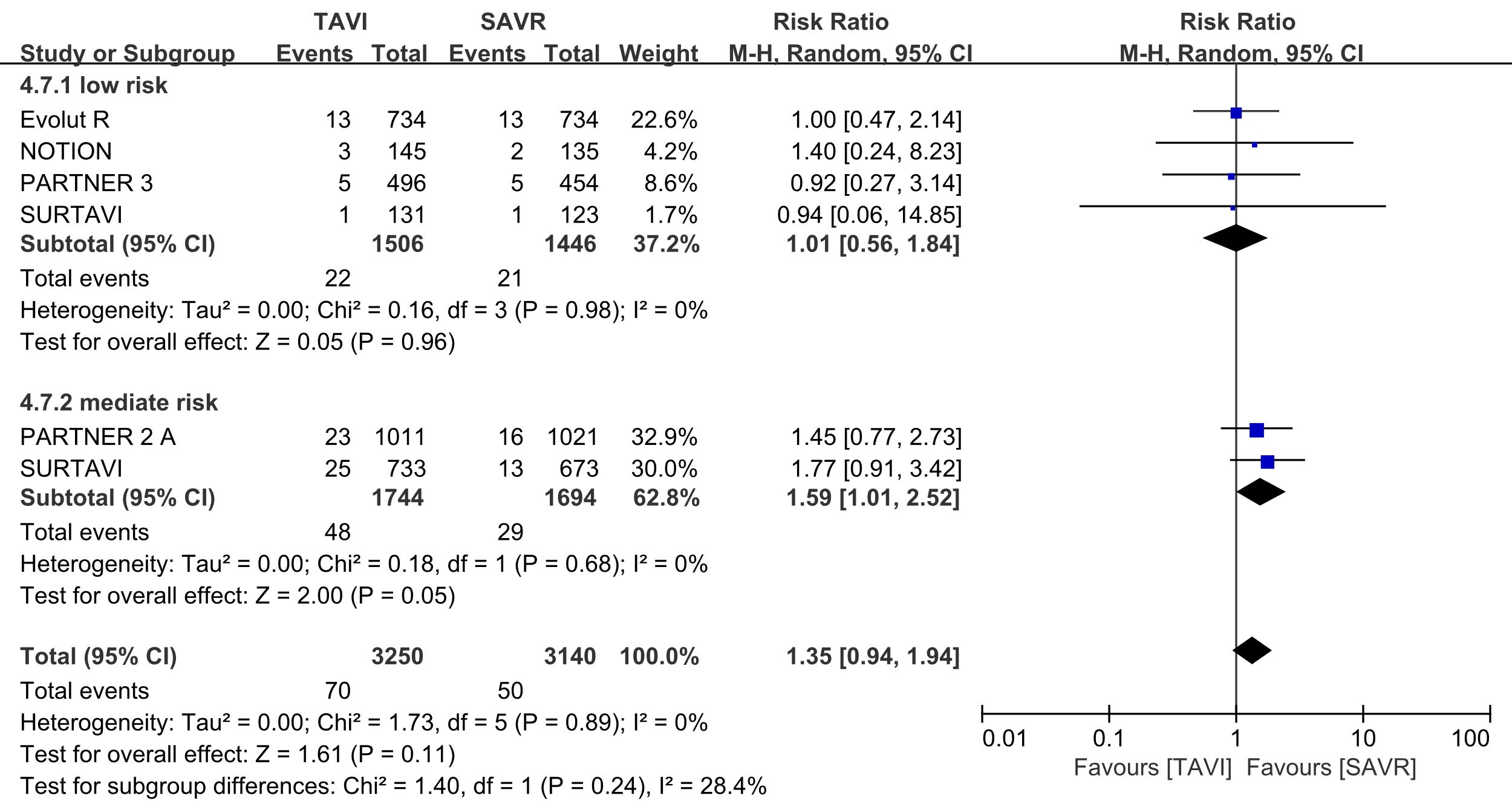


### Fig G: Forest plot for endocarditis

###
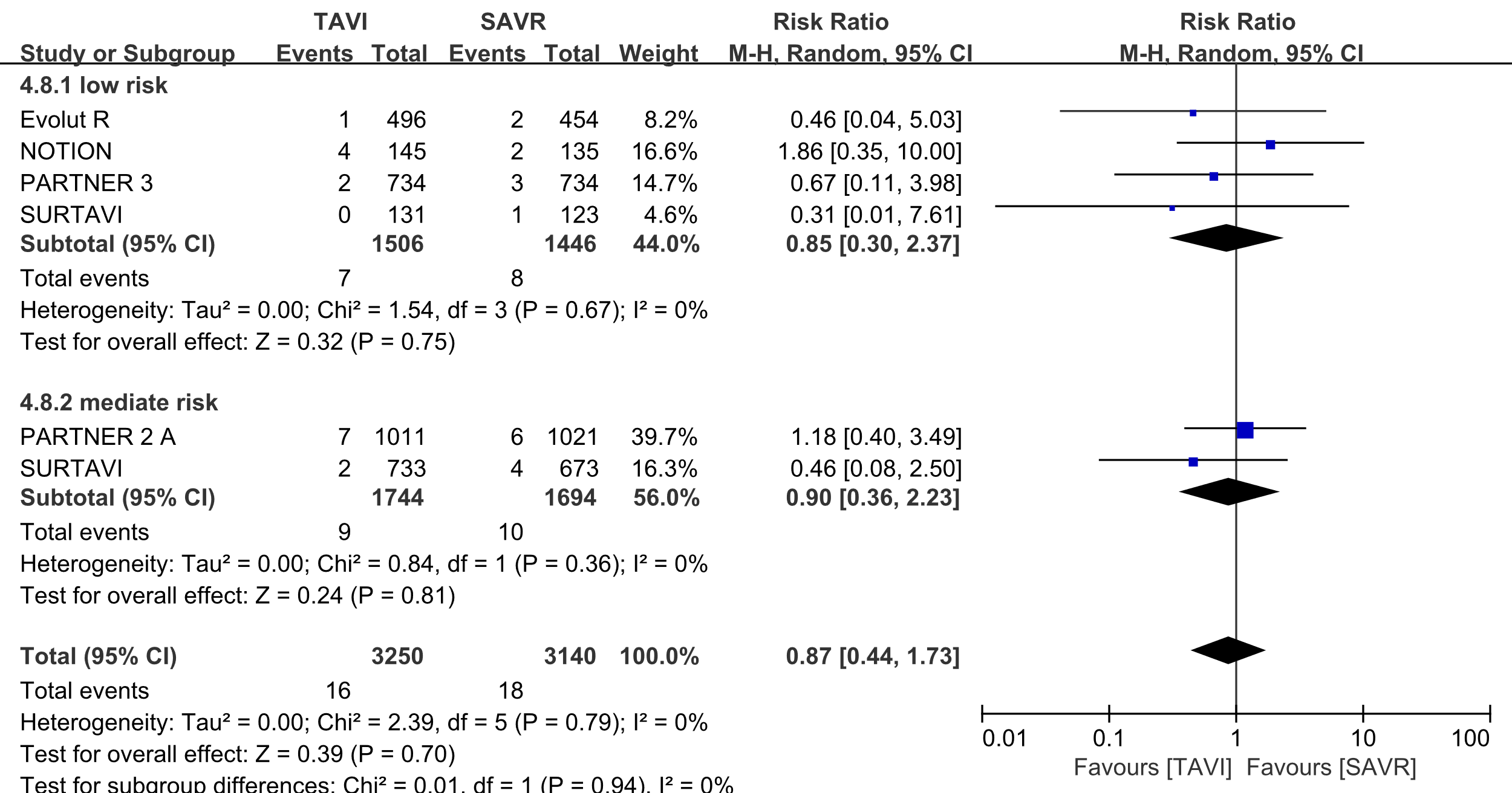


### Fig H: Forest plot for acute kidney injury stage 2 or 3

###
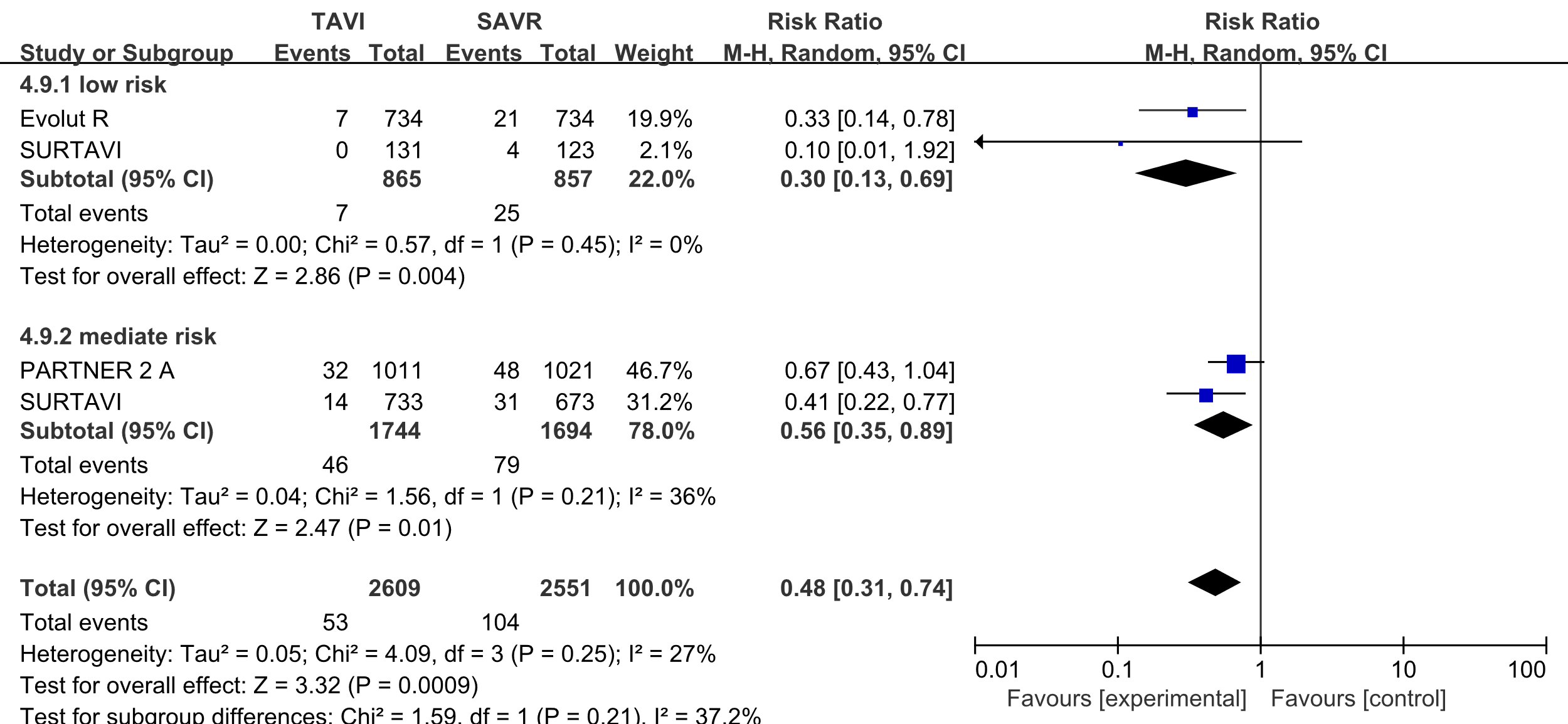


### Fig I: Forest plot for life-threatening or disabling bleeding

###
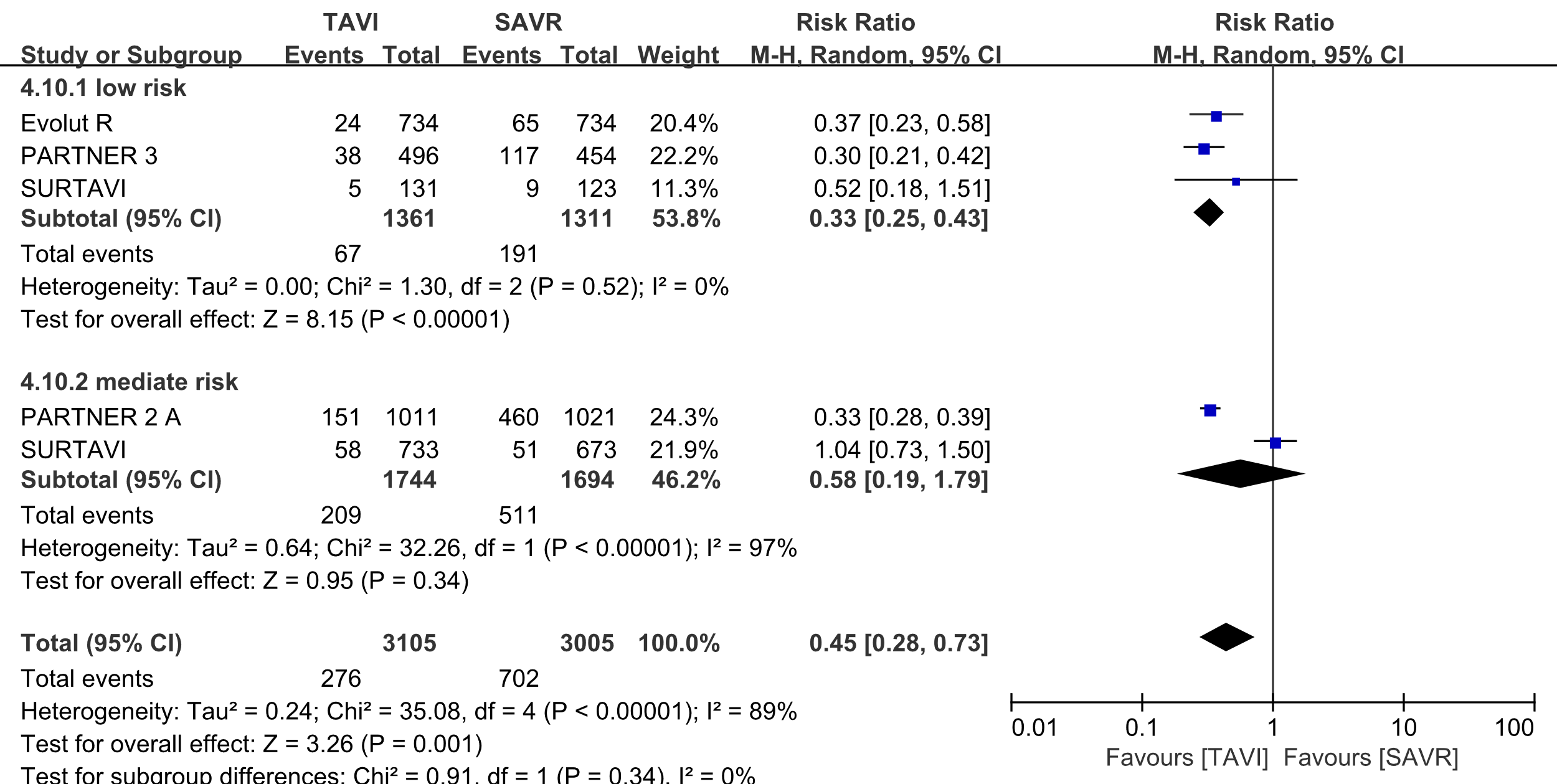


### Fig J: Forest plot for myocardial infarction

###
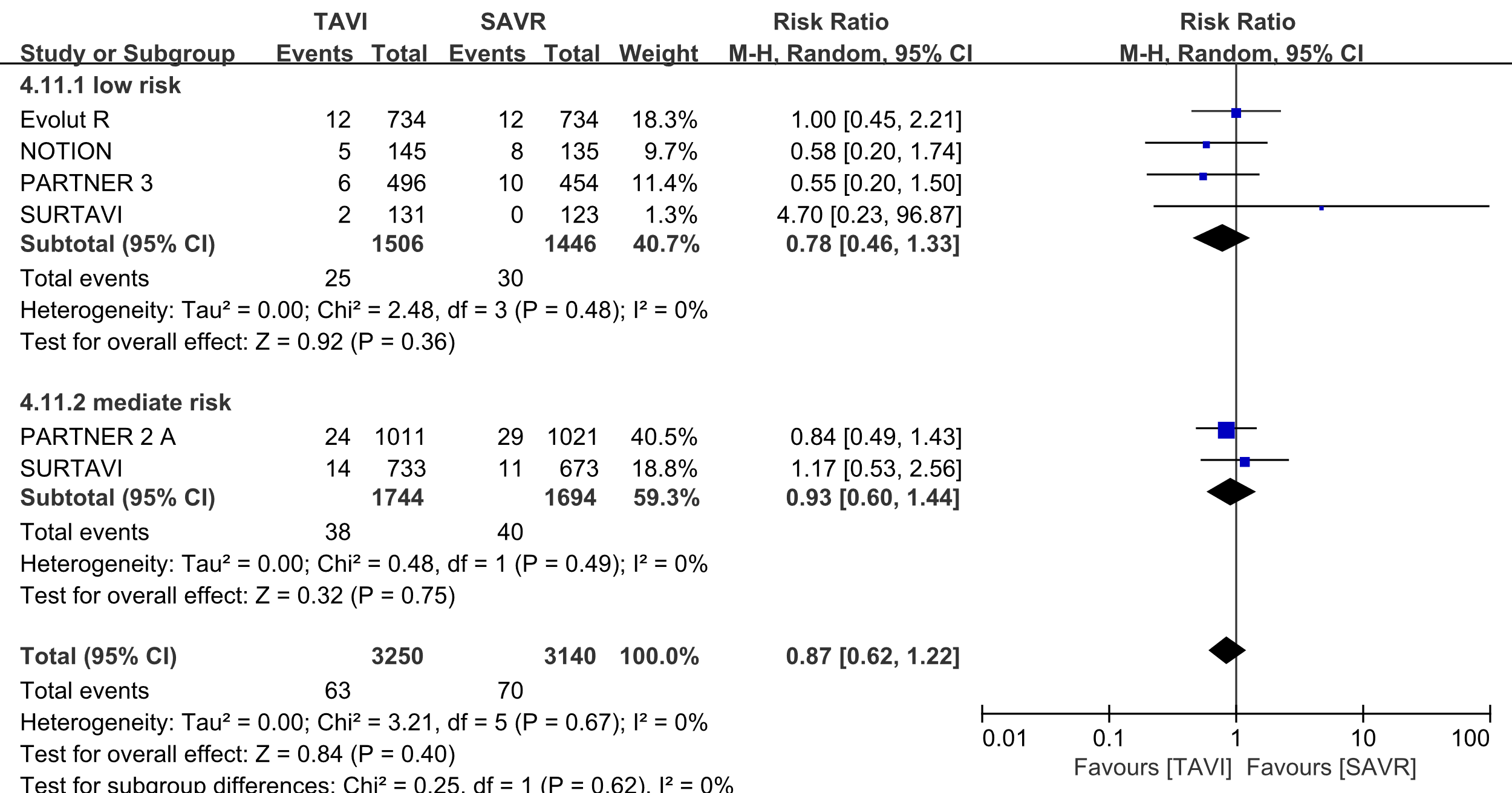


### References:

1. Smith CR, Leon MB, Mack MJ, et al. Transcatheter versus surgical aortic-valve replacement in high-risk patients. *The New England journal of medicine.* 2011;364(23):2187-2198.

2. Nielsen HH, Klaaborg KE, Nissen H, et al. A prospective, randomised trial of transapical transcatheter aortic valve implantation vs. surgical aortic valve replacement in operable elderly patients with aortic stenosis: the STACCATO trial. *EuroIntervention : journal of EuroPCR in collaboration with the Working Group on Interventional Cardiology of the European Society of Cardiology.* 2012;8(3):383-389.

3. Adams DH, Popma JJ, Reardon MJ, et al. Transcatheter aortic-valve replacement with a self-expanding prosthesis. *The New England journal of medicine.* 2014;370(19):1790-1798.

4. Feldman TE, Reardon MJ, Rajagopal V, et al. Effect of Mechanically Expanded vs Self-Expanding Transcatheter Aortic Valve Replacement on Mortality and Major Adverse Clinical Events in High-Risk Patients With Aortic Stenosis: The REPRISE III Randomized Clinical TrialEffect of Mechanically Expanded vs Self-Expanding TAVR for Aortic StenosisEffect of Mechanically Expanded vs Self-Expanding TAVR for Aortic Stenosis. *Jama.* 2018;319(1):27-37.

5. Tarantini G, Nai Fovino L, Gersh BJ. Transcatheter aortic valve implantation in lower-risk patients: what is the perspective? *European heart journal.* 2018;39(8):658-666.

6. Reardon MJ, Van Mieghem NM, Popma JJ, et al. Surgical or Transcatheter Aortic-Valve Replacement in Intermediate-Risk Patients. *The New England journal of medicine.* 2017;376(14):1321-1331.

7. Serruys PW, Modolo R, Reardon M, et al. One-year outcomes of patients with severe aortic stenosis and an STS PROM of less than three percent in the SURTAVI trial. *EuroIntervention : journal of EuroPCR in collaboration with the Working Group on Interventional Cardiology of the European Society of Cardiology.* 2018;14(8):877-883.
